# The use of teleconsultation and technology by the Aravind Eye Care System, India

**Published:** 2022-06-07

**Authors:** R Kim, Chitaranjan Mishra, Sagnik Sen

**Affiliations:** 1 Chief Medical Officer: Retina Vitreous Services; Director: Informational Technology & Systems, Aravind Eye Hospital, Madurai, India.; 2 Medical Consultant: Department of Vitreo-Retina, Aravind Eye Hospital, Madurai, India.; 3 Fellow: Department of Vitreo-Retina, Aravind Eye Hospital, Madurai, India.

**Teleconsultation in vision centres, as a part of teleophthalmology services, is a critical component of primary patient care at the Aravind Eye Care System in India.**

**Figure:**
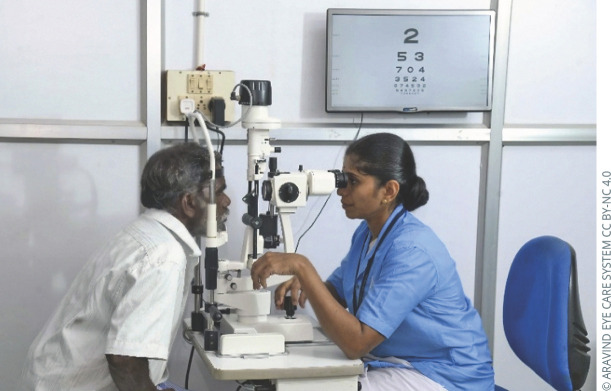
The vision technician examines a patient at the vision centre before beginning a teleconsultation with the ophthalmologist at Aravind eye hospital. **INDIA**

Teleconsultation services are a key element of teleophthalmology. Teleophthalmology is a coordinated eye health care approach that connects patients and health care providers via information and communications technology (ICT) to enable health care to reach remote and underserved areas.

In India, the Aravind Eye Care System (AECS) has been deploying teleophthalmology at the primary and secondary levels of eye care. The Aravind Teleophthalmology Network (ATN) provides accessible and affordable eye care service to people in remote areas using advanced communications technology, saving time and money that would otherwise be spent on travel.^1^

## Teleconsultation in vision centres: primary level eye care

Vision centres are primary eye care facilities based in rural and semi-rural communities. The centres, which were started in India in 2004, have grown in number and become an important part of eye care services in both the government and private sectors.

Each vision centre is managed by a trained ophthalmic technician and a coordinator. It has basic ophthalmic equipment, such as a slit lamp, an applanation tonometer, a trial lens set for refraction, and two computers with broadband internet connectivity.

**Figure:**
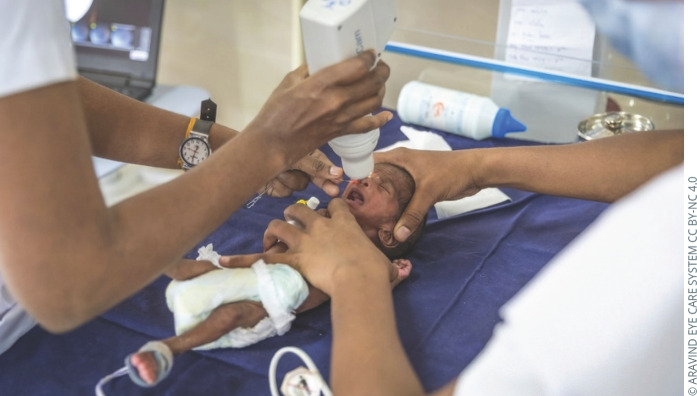
Telescreening for retinopathy of prematurity at secondary level. The images recorded during the scan are shared with specialist peadiatric ophthalmologists at tertiary or specialist hospitals. **INDIA**

During a teleconsultation, the ophthalmic technician examines the patient, records the refractive error and the anterior segment and fundus findings (on 90D examination), and documents the findings in a secure electronic medical record (EMR) which can be accessed securely by ophthalmologists working at the base hospital. Next, during a video call with the patient and the ophthalmic technician, an ophthalmologist at the base hospital views the EMR along with the images and any additional information provided by the technician. The ophthalmologist then enters her or his advice in the EMR and discusses the next steps with the patient.

Aravind vision centres together carry out more than 2,800 teleconsultations a day, and this model has been replicated by others in many states in India as well as across Bangladesh. Data from the AECS vision centre registry indicate that 15–17% of patients seen at AECS vision centres had to be referred to a tertiary centre.

## Technology in secondary level eye care

At the secondary level, technology can be used to support screening for conditions that affect the back of the eye, such as diabetic retinopathy (DR) and retinopathy of prematurity (ROP). Early diagnosis and treatment of these conditions can prevent needless blindness; however, not all secondary or district hospitals have retinal specialists who are able to to diagnose these conditions.

The availability of tools based on artificial intelligence (AI) that can analyse retinal images has made the diagnosis of DR quicker and simpler. For ROP, retinal scans taken in neonatal intensive care units can be sent to specialists at tertiary hospital level for analysis and identification of ROP.

## Barriers

When implementing teleophthalmology, the major barriers are the initial cost of investment, difficulties capturing high quality digital images, the shortage of trained and dedicated health care and teleophthalmology personnel, and concerns about the privacy and security of patient data.
